# RETRACTION: Effect of Salpingectomy Versus Tubal Ligation on Postoperative Wound Infection in Patients: A Meta‐Analysis

**DOI:** 10.1111/iwj.70614

**Published:** 2025-04-21

## Abstract

**RETRACTION:** LiM. and LvJ. , “Effect of Salpingectomy Versus Tubal Ligation on Postoperative Wound Infection in Patients: A Meta‐Analysis,” International Wound Journal 21, no. 1 (2024): e14543, 10.1111/iwj.14543.38272821 PMC10805532

The above article, published online on 23 January 2024, in Wiley Online Library (http://onlinelibrary.wiley.com/), has been retracted by agreement between the journal Editor in Chief, Professor Keith Harding; and John Wiley & Sons Ltd. Following an investigation by the publisher, all parties have concluded that this article was accepted solely on the basis of a compromised peer review process. In addition, further investigation by the publisher found that the Methods lacked sufficient details for reproducibility, that the research question was insufficiently supported by the Results presented, and that the Discussion and Conclusion sections were not supported by the supplied references. The editors have therefore decided to retract the article. The authors did not respond to our notice regarding the retraction.

**RETRACTION:** M. Li and J. Lv , “Effect of Salpingectomy Versus Tubal Ligation on Postoperative Wound Infection in Patients: A Meta‐Analysis,” International Wound Journal 21, no. 1 (2024): e14543, 10.1111/iwj.14543.38272821 PMC10805532

The above article, published online on 23 January 2024, in Wiley Online Library (http://onlinelibrary.wiley.com/), has been retracted by agreement between the journal Editor in Chief, Professor Keith Harding; and John Wiley & Sons Ltd. Following an investigation by the publisher, all parties have concluded that this article was accepted solely on the basis of a compromised peer review process. In addition, further investigation by the publisher found that the Methods lacked sufficient details for reproducibility, that the research question was insufficiently supported by the Results presented, and that the Discussion and Conclusion sections were not supported by the supplied references. The editors have therefore decided to retract the article. The authors did not respond to our notice regarding the retraction.

